# Supplementary material for: STAT1 regulates immune-mediated intestinal stem cell proliferation and epithelial regeneration
Source: Nat Commun. 2025 Jan 2;16:138. doi: 10.1038/s41467-024-55227-5 (PMC11697299; doi:10.1038/s41467-024-55227-5)
Supplement: Supplementary file 1 — Supplementary Information [file 41467_2024_55227_MOESM1_ESM.pdf]

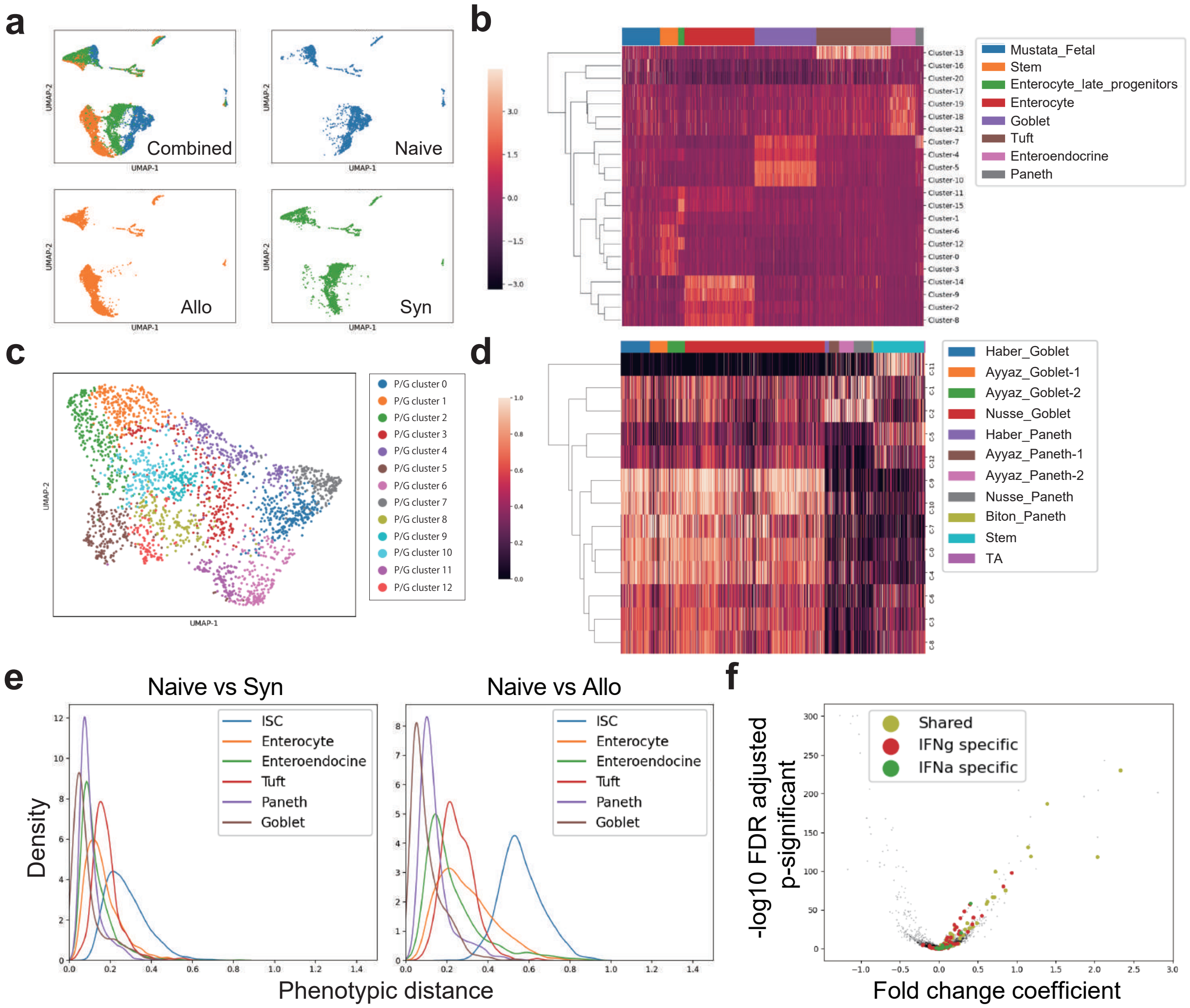

Supplementary Figure 1. Single cell RNA sequencing of intestinal crypts after MHC-mismatched allo-BMT. Ileal crypts were isolated from healthy B6 mice (Naive) or from transplant recipients five days after B6-into-B6 syngeneic (Syn) or B10.Br-into-B6 allogeneic (Allo) BMT, then analyzed by scRNA-seq. **a** UMAP indicating the experimental groups of origin for each cluster. **b** Expression heatmap of cell-type specific genes. **c** UMAP plots after sub-clustering of Paneth/goblet cell population (Original clusters 4, 5, 7 and 10). **d** Expression heatmap of cell-type specific genes in Paneth/goblet cell populations. **e** Histograms showing phenotypic distances between Naive and Allo populations (left) or Naive and Syn populations (right) in units of log-normalized gene expression. **f** Volcano plot showing IFN response genes and other differentially expressed genes in ISCs between Syn and Allo samples.

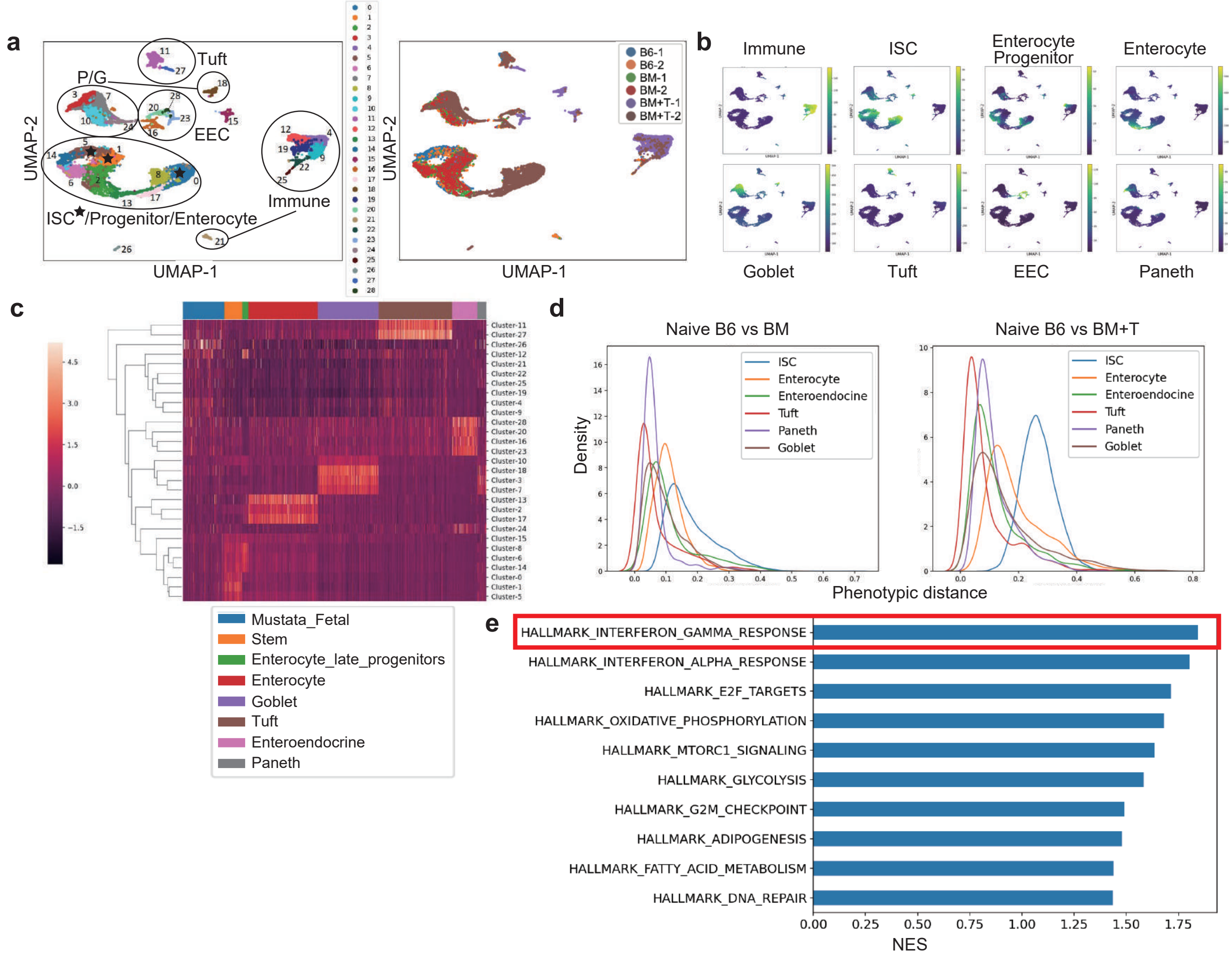

Supplementary Figure 2. Single cell RNA sequencing of intestinal crypts after MHC-matched allogeneic BMT. Small intestine crypts were isolated from healthy B6 mice (B6) or from LP-into-B6 MHC-matched BMT recipients, 10 days after receiving donor marrow alone (BM) or marrow and T cells (BM+T), then analyzed by scRNA-seq. **a** UMAP plots indicating distinct clusters (left) or the experimental groups from which each cluster is derived (right). **b** UMAP plots indicating gene expression profiles for cellular subtypes isolated from intestinal crypts. **c** Expression heatmap of cell-type specific genes. **d** Histograms showing phenotypic distances between B6 and BM samples (left) or B6 and BM+T samples (right) in units of log-normalized gene expression. **e** GSEA analysis of differentially expressed genes in ISCs from BM and BM+T samples; NES: normalized enrichment score.

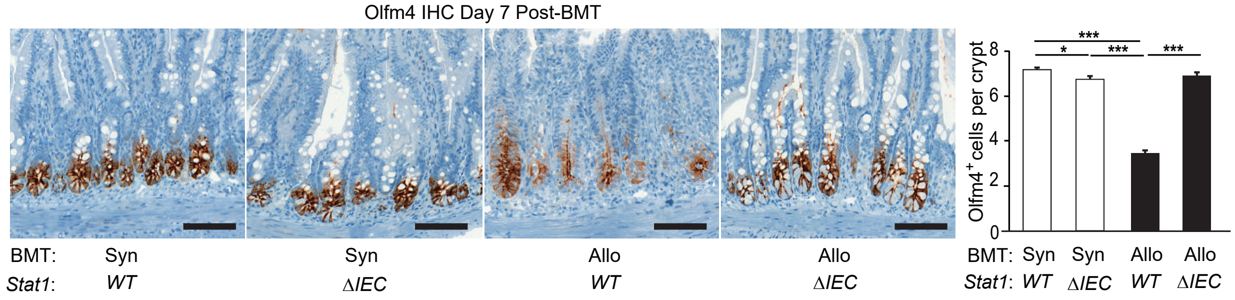

Supplementary Figure 3. ISCs day 7 post-BMT. Allogeneic B10.Br-into-B6 (Allo) or syngeneic B6-into-B6 (Syn) BMT using *Stat1*$^{fl/fl}$x*Villin-Cre* (*Stat1*$^{\Delta IEC}$) or Cre-negative *Stat1*$^{fl/fl}$ (*Stat1*^{WT}) littermate controls. Ileum was harvested seven days post-BMT. Shown are representative staining and quantification of Olfm4^{+} ISCs (n = 417 Syn *Stat1*^{WT}, 312 Syn *Stat1*$^{\Delta IEC}$, 1185 Allo *Stat1*^{WT}, 1049 Allo *Stat1*$^{\Delta IEC}$ crypts; scale bars = 50μm). Graphical data represent the mean and s.e.m.; comparisons performed with one-way ANOVA multiple comparison testing; Syn *Stat1*^{WT} vs Syn *Stat1*$^{\Delta IEC}$, $p = 0.0435$; * $p < 0.05$, *** $p < 0.001$. The exact *p*-values are $p < 0.001$ unless specified otherwise.

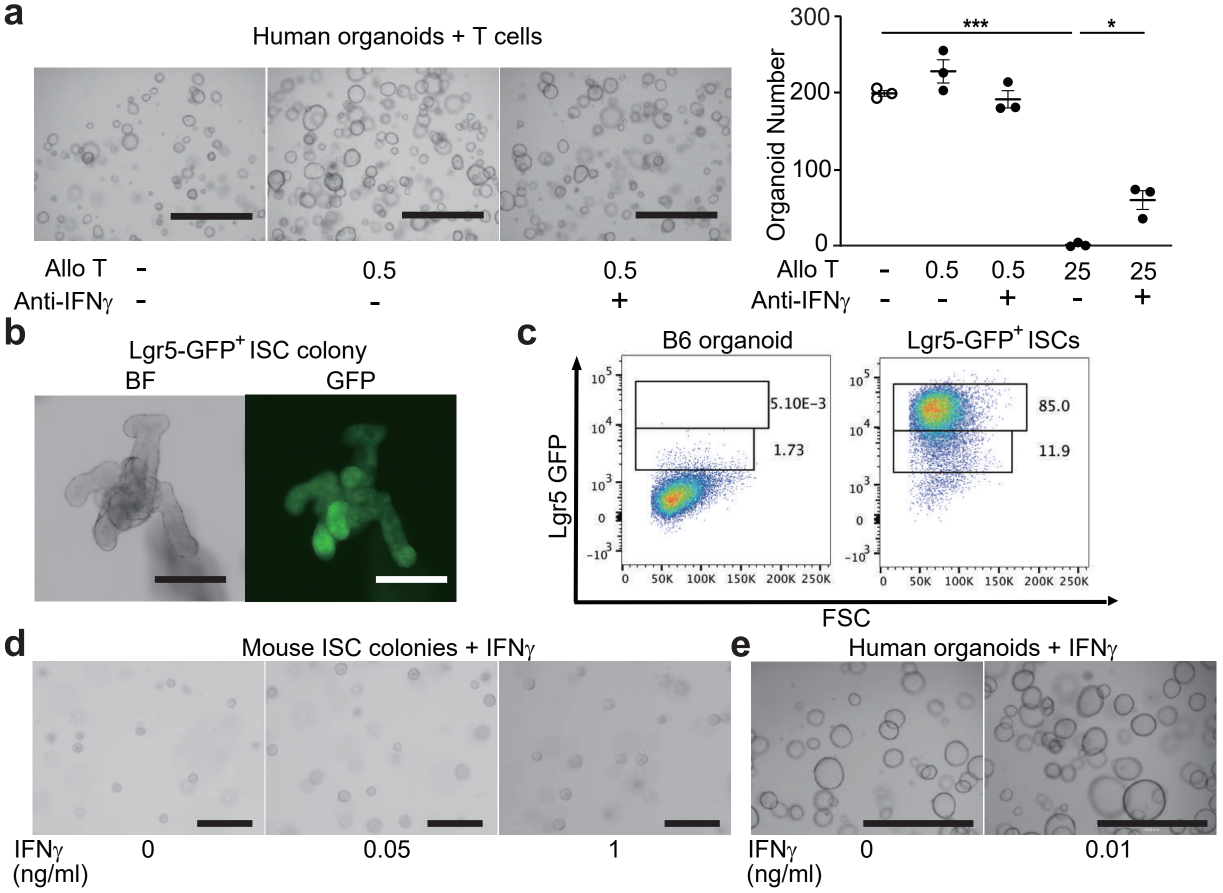

Supplementary Figure 4. T-cell-derived IFNγ stimulates mouse and human epithelial regeneration *ex vivo*. **a** Representative images and number of human duodenal organoids (passaged from 500 organoids per well) cultured with human allogeneic T cells (x10^3/well) +/- anti-IFNγ neutralizing antibodies (culture day 7, n = 3 wells per group); scale bars = 1000 μm. Graphical data indicate the mean and s.e.m.; analyzed by one-way ANOVA multiple comparison testing; No allo T vs 25 Allo T, $p < 0.001$; 25 Allo T vs +anti-IFNγ, $p = 0.0153$; * $p < 0.05$, *** $p < 0.001$. **b** Representative Lgr5-GFP$^+$ ISC colony images (culture day 6); left, bright field; right, GFP; scale bars = 200 μm. **c** FACS analysis of Lgr5-GFP^{high} ISCs in negative control wild-type B6 SI organoids (left) and in SI ISC colonies (right); culture day 6. **d** Representative images of SI ISC colonies cultured with IFNγ for 3 days; scale bars = 200 μm. **e** Representative images of human duodenal organoids cultured +/- IFNγ (culture day 7); scale bars = 1000 μm. a-e are representative of two independent experiments.

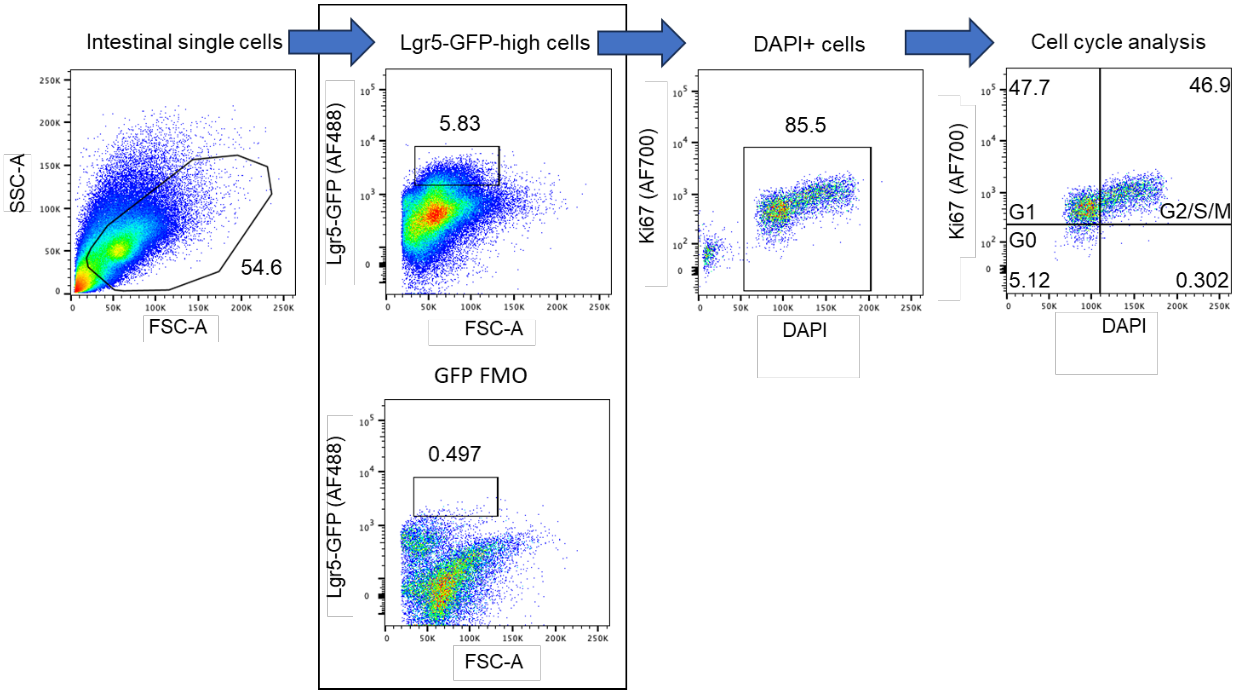

Supplementary Figure 5. Flow-cytometry-based cell cycle analysis. Analysis was performed after gating Lgr5-GFP^{high} cells and excluding DAPI^{+} cells. The GFP FMO panel indicates the GFP-negative "fluorescence minus one" control for setting the GFP^{high} gate.

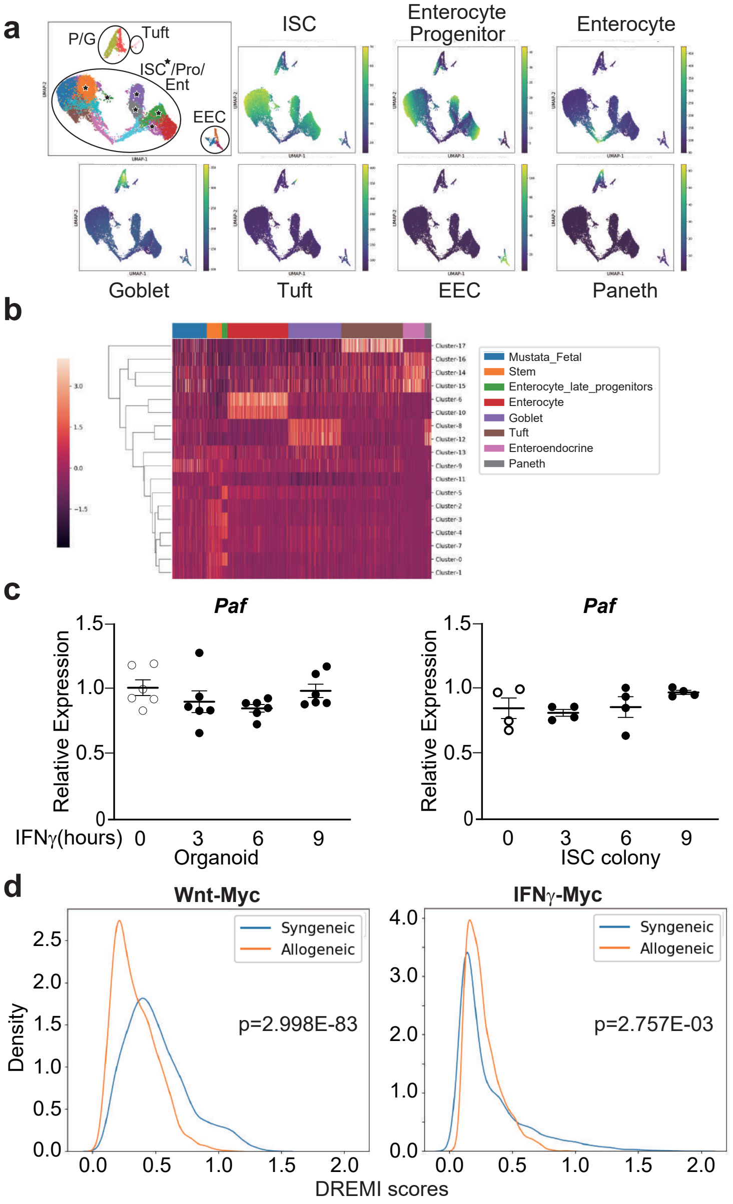

Supplementary Figure 6. Epithelial cultures exposed to IFNγ. a-b Single cell RNA sequencing of B6 SI intestinal organoids treated with IFNγ (0.05 ng/ml, culture day 5). **a** UMAP plots showing annotated clusters (upper left) and gene expression profiles for subtypes of intestinal epithelial cells. **b** Expression heatmap of cell-type specific genes. **c** *Paf* gene expression in mouse SI organoids (left, n = 6 wells/group) and ISC colonies (right, n = 4 wells/group) cultured with IFNγ (1 ng/ml). Graphical data indicate the mean and s.e.m. and are representative of two independent experiments. Kruskal-Wallis multiple comparison testing indicated no significant differences. **d** DREMI analysis demonstrating strength of gene-gene interactions between Myc and Wnt target genes and between Myc and IFNγ target genes based on scRNA-seq analysis of ISC clusters on

day 5 after B6-into-B6 syn-BMT or B10.Br-into-B6 allo-BMT, indicating greater association between Myc and Wnt pathway targets after syngeneic BMT (no GVHD) and greater association between Myc and IFNγ pathways targets after allogeneic BMT (GVHD setting); *p*-values computed using RankSum tests.

Supplementary Data 1. Differentially expressed ISC genes after allogeneic vs. syngeneic BMT.

(Separate File)

Supplementary Data 2. Genes correlating with *Stat1* expression in ISCs after allogeneic BMT.

(Separate File)

Supplementary Data 3. Wnt and Myc pathway gene associations in ISCs after syngeneic BMT.

(Separate File)

Supplementary Data 4. Wnt and Myc pathway gene associations in ISCs after allogeneic BMT.

(Separate File)

Supplementary Data 5. IFNγ and Myc pathway gene associations in ISCs after syngeneic BMT.

(Separate File)

Supplementary Data 6. IFNγ and Myc pathway gene associations in ISCs after allogeneic BMT.

(Separate File)

Supplementary Table 1. List of antibodies used.

ANTIBODY	SOURCE	IDENTIFIER
Donkey anti-Goat IgG (H+L) Cross-Adsorbed Secondary Antibody	Invitrogen	A-11056
Donkey anti-Rabbit IgG (H+L) Highly Cross-Adsorbed Secondary Antibody	Invitrogen	A-31573
Rat anti-IFNγ	Bio X Cell	BP0055
Mouse anti-CD3	Biolegend	300414
Mouse anti-CD28	Biolegend	302914
Mouse anti-IFNγ	eBioecience	16-7318-85
Mouse anti-Ki-67	BD Pharmingen	561277
Hamster anti-CD3	BD Pharmingen	553058
Hamster anti-CD28	BD Pharmingen	557393
Rabbit anti-c-Myc	Abcam	ab32072
Rabbit normal IgG	Cell Signaling	2729
Rabbit anti-Ki67	Cell Signaling	12202S
Rabbit anti-Olfm4	Cell Signaling	39141S
Rabbit anti-STAT1	Cell Signaling	9172
Goat anti-c-Myc	R&D systems	AF3696
Biotinylated goat anti-rabbit IgG	Vector Labs	PK-6101
