# Supplementary material for: STAT1 regulates immune-mediated intestinal stem cell proliferation and epithelial regeneration
Source: Nat Commun. 2025 Jan 2;16:138. doi: 10.1038/s41467-024-55227-5 (PMC11697299; doi:10.1038/s41467-024-55227-5)
Supplement: Supplementary file 3 — Description of Additional Supplementary Files [file 41467_2024_55227_MOESM3_ESM.pdf]

Description of Additional Supplementary Files

Supplementary Data 1. Differentially expressed ISC genes after allogeneic vs. syngeneic BMT.

Supplementary Data 2. Genes correlating with Stat1 expression in ISCs after allogeneic BMT.

Supplementary Data 3. Wnt and Myc pathway gene associations in ISCs after syngeneic BMT.

Supplementary Data 4. Wnt and Myc pathway gene associations in ISCs after allogeneic BMT.

Supplementary Data 5. IFNg and Myc pathway gene associations in ISCs after syngeneic BMT.

Supplementary Data 6. IFNg and Myc pathway gene associations in ISCs after allogeneic BMT.
