# Supplementary material for: STAT1 regulates immune-mediated intestinal stem cell proliferation and epithelial regeneration
Source: Nat Commun. 2025 Jan 2;16:138. doi: 10.1038/s41467-024-55227-5 (PMC11697299; doi:10.1038/s41467-024-55227-5)
Supplement: Supplementary file 10 — Reporting Summary [file 41467_2024_55227_MOESM10_ESM.pdf]

nature portfolio

Corresponding author(s): Alan M. Hanash

Last updated by author(s): Oct 1, 2024

Reporting Summary

Nature Portfolio wishes to improve the reproducibility of the work that we publish. This form provides structure for consistency and transparency in reporting. For further information on Nature Portfolio policies, see our Editorial Policies and the Editorial Policy Checklist.

Statistics

For all statistical analyses, confirm that the following items are present in the figure legend, table legend, main text, or Methods section.

n/a	Confirmed	
☐	☒	The exact sample size (*n*) for each experimental group/condition, given as a discrete number and unit of measurement
☐	☒	A statement on whether measurements were taken from distinct samples or whether the same sample was measured repeatedly
☐	☒	The statistical test(s) used AND whether they are one- or two-sided<br>*Only common tests should be described solely by name; describe more complex techniques in the Methods section.*
☒	☐	A description of all covariates tested
☒	☐	A description of any assumptions or corrections, such as tests of normality and adjustment for multiple comparisons
☐	☒	A full description of the statistical parameters including central tendency (e.g. means) or other basic estimates (e.g. regression coefficient) AND variation (e.g. standard deviation) or associated estimates of uncertainty (e.g. confidence intervals)
☐	☒	For null hypothesis testing, the test statistic (e.g. *F*, *t*, *r*) with confidence intervals, effect sizes, degrees of freedom and *P* value noted<br>*Give P values as exact values whenever suitable.*
☒	☐	For Bayesian analysis, information on the choice of priors and Markov chain Monte Carlo settings
☒	☐	For hierarchical and complex designs, identification of the appropriate level for tests and full reporting of outcomes
☒	☐	Estimates of effect sizes (e.g. Cohen's *d*, Pearson's *r*), indicating how they were calculated

Our web collection on statistics for biologists contains articles on many of the points above.

Software and code

Policy information about availability of computer code

Data collection	FACSDiva version 6.1.2. (BD Biosciences), Zen (Zaiss) version2.0, Gen 5 version 3.10 (Bio Tek)
Data analysis	FlowJO software version 9.9.4 (TreeStar), Graph Pad Prism version 10 (Graph Pad Software), ZEN software version 2.0 (Zeiss), QuantStudio™ Real-Time PCR Software version 1.7. (Thermo Fisher Scientific), ImageJ version 1.52, Panoramic Viewer version 1.15.3, Amira 6.0.1 image reconstruction software (FEI)

For manuscripts utilizing custom algorithms or software that are central to the research but not yet described in published literature, software must be made available to editors and reviewers. We strongly encourage code deposition in a community repository (e.g. GitHub). See the Nature Portfolio guidelines for submitting code & software for further information.

Data

Policy information about availability of data

All manuscripts must include a data availability statement. This statement should provide the following information, where applicable:

- Accession codes, unique identifiers, or web links for publicly available datasets
- A description of any restrictions on data availability
- For clinical datasets or third party data, please ensure that the statement adheres to our policy

The single cell RNA sequencing data used in this study have been deposited in the Gene Expression Omnibus under the accession number GSE276126.

Research involving human participants, their data, or biological material

Policy information about studies with human participants or human data. See also policy information about sex, gender (identity/presentation), and sexual orientation and race, ethnicity and racism.

Reporting on sex and gender	In vitro experiments were performed with material from both male and female mice. Transplants using wild-type recipients were generally performed with female mice. Transplants using transgenic recipients were performed using a mix of male and female mice.
Reporting on race, ethnicity, or other socially relevant groupings	N/A
Population characteristics	Biobanked organoids from healthy donors were used. The donors were originally included in a biobanking protocol (details in methods section) when undergoing a clinically required diagnostic endoscopy procedure, only after written informed consent and institutional ethical committee approval. All healthy controls had been investigated for celiac disease, but were found to have normal histopathology. They consented for the establishment, maintenance and freezing of intestinal organoid lines and their use in laboratory studies investigating epithelial and inflammatory intestinal disease.
Recruitment	See above.
Ethics oversight	The protocol was reviewed and approved by the review board of the UMC Utrecht, the Netherlands (protocol STEM study, METC 10-402/K).

Note that full information on the approval of the study protocol must also be provided in the manuscript.

Field-specific reporting

Please select the one below that is the best fit for your research. If you are not sure, read the appropriate sections before making your selection.

☒ Life sciences ☐ Behavioural & social sciences ☐ Ecological, evolutionary & environmental sciences

For a reference copy of the document with all sections, see nature.com/documents/nr-reporting-summary-flat.pdf

Life sciences study design

All studies must disclose on these points even when the disclosure is negative.

Sample size	At least three to five mice per group were used in each in vivo experiment. Three to four biological replicates defined by individual wells were used for in vitro analysis. The precise numbers of animals and wells are reported in the figure legends.
Data exclusions	This information is included in the Methods.
Replication	Experiments were repeated so that our data are typically based on two to three independent experiments. The precise number of repeats are reported in the figure legends.
Randomization	Genetically identical mice were purchased from the vendor, and then randomly distributed across different cages and groups by a non-biased technician who had no insight or information about the purpose or details of the experiment.
Blinding	See above.

Reporting for specific materials, systems and methods

We require information from authors about some types of materials, experimental systems and methods used in many studies. Here, indicate whether each material, system or method listed is relevant to your study. If you are not sure if a list item applies to your research, read the appropriate section before selecting a response.

Materials & experimental systems

n/a	Involved in the study	
☐	☒	Antibodies
☐	☒	Eukaryotic cell lines
☒	☐	Palaeontology and archaeology
☐	☒	Animals and other organisms
☐	☒	Clinical data
☒	☐	Dual use research of concern
☒	☐	Plants

Methods

n/a	Involved in the study	
☒	☐	ChIP-seq
☐	☒	Flow cytometry
☒	☐	MRI-based neuroimaging

Antibodies

Antibodies used	All antibody information including clone numbers and company source are provided on the table in the Methods.
Validation	All antibodies were validated according to the manufacture's instruction or relevant citations.

Eukaryotic cell lines

Policy information about cell lines and Sex and Gender in Research

Cell line source(s)	This information is included in the Methods.
Authentication	N/A
Mycoplasma contamination	All the cell lines were tested Mycoplasma contamination, and the results were all negative.
Commonly misidentified lines (See ICLAC register)	N/A

Animals and other research organisms

Policy information about studies involving animals; ARRIVE guidelines recommended for reporting animal research, and Sex and Gender in Research

Laboratory animals	This information is included in the Methods.
Wild animals	N/A
Reporting on sex	See Methods.
Field-collected samples	N/A
Ethics oversight	All animal experiments were performed in accordance with the guidelines of the Memorial Sloan Kettering Cancer Center (MSKCC) Institutional Animal Care and Use Committee.

Note that full information on the approval of the study protocol must also be provided in the manuscript.

Clinical data

Policy information about clinical studies

All manuscripts should comply with the ICMJE guidelines for publication of clinical research and a completed CONSORT checklist must be included with all submissions.

Clinical trial registration	N/A
Study protocol	N/A
Data collection	N/A
Outcomes	N/A

Plants

Seed stocks	N/A
Novel plant genotypes	N/A
Authentication	N/A

Flow Cytometry

Plots

Confirm that:

☒ The axis labels state the marker and fluorochrome used (e.g. CD4-FITC).

☒ The axis scales are clearly visible. Include numbers along axes only for bottom left plot of group (a 'group' is an analysis of identical markers).

☒ All plots are contour plots with outliers or pseudocolor plots.

☒ A numerical value for number of cells or percentage (with statistics) is provided.

Methodology

Sample preparation	This information is included in the Methods.
Instrument	LSR-II or X-50 cytometer (BD Biosciences)
Software	The data were collected with FACSDiva version 6.1.2 (BD Biosciences), and analyzed with FlowJo software version 9.9.4 (Treestar).
Cell population abundance	Sort-purification was carried out using FACSAria cell sorter (BD Bioscience).
Gating strategy	The gating was performed as described previously (Takashima et al, Science Immunology, 2019). In brief, after gating single cell population in the FSC vs SSC plots and excluding DAPI+ dead cells and CD45+ cells, ISCs were identified as Lgr5-GFP high cells.

☒ Tick this box to confirm that a figure exemplifying the gating strategy is provided in the Supplementary Information.
